# Comparison of the Operative Outcomes and Learning Curves between Laparoscopic and Robotic Gastrectomy for Gastric Cancer

**DOI:** 10.1371/journal.pone.0111499

**Published:** 2014-10-31

**Authors:** Kuo-Hung Huang, Yuan-Tzu Lan, Wen-Liang Fang, Jen-Hao Chen, Su-Shun Lo, Anna Fen-Yau Li, Shih-Hwa Chiou, Chew-Wun Wu, Yi-Ming Shyr

**Affiliations:** 1 Division of General Surgery, Department of Surgery, Taipei Veterans General Hospital, Taipei City, Taiwan; 2 Department of Pathology, Taipei Veterans General Hospital, Taipei City, Taiwan; 3 Division of Colon & Rectal Surgery, Department of Surgery, Taipei Veterans General Hospital, Taipei City, Taiwan; 4 School of Medicine, National Yang-Ming University, Taipei City, Taiwan; 5 Institute of Clinical Medicine, School of Medicine, National Yang-Ming University, Taipei City, Taiwan; 6 En Chu Kong Hospital, New Taipei City, Taiwan; 7 National Yang-Ming University Hospital, Yilan City, Taiwan; 8 Department of Medical Research and Education, Taipei Veterans General Hospital, Taipei City, Taiwan; 9 Institute of Pharmacology, National Yang-Ming University, Taipei City, Taiwan; UT MD Anderson Cancer Center, United States of America

## Abstract

**Background:**

Minimally invasive surgery, including laparoscopic and robotic gastrectomy, has become more popular in the treatment of gastric cancer. However, few studies have compared the learning curves between laparoscopic and robotic gastrectomy for gastric cancer.

**Methods:**

Data were prospectively collected between July 2008 and Aug 2014. A total of 145 patients underwent minimally invasive gastrectomy for gastric cancer by a single surgeon, including 73 laparoscopic and 72 robotic gastrectomies. The clinicopathologic characteristics, operative outcomes and learning curves were compared between the two groups.

**Results:**

Compared with the laparoscopic group, the robotic group was associated with less blood loss and longer operative time. After the surgeon learning curves were overcome for each technique, the operative outcomes became similar between the two groups except longer operative time in the robotic group. After accumulating more cases of robotic gastrectomy, the operative time in the laparoscopic group decreased dramatically.

**Conclusions:**

After overcoming the learning curves, the operative outcomes became similar between laparoscopic and robotic gastrectomy. The experience of robotic gastrectomy could affect the learning process of laparoscopic gastrectomy.

## Introduction

Minimally invasive gastrectomy is becoming a widely accepted procedure, especially in Asian countries. Laparoscopic gastrectomy offers improved early postoperative outcomes and improved long-term oncologic outcomes that are comparable to those that are achieved with open gastrectomy [1]–[3].

The use of robotic surgery can achieve precise lymph node dissection in gastric cancer, afford surgeons a more comfortable operating environment and decrease mental stress during the surgery. Furthermore, for surgeons with laparoscopic surgery experience, fewer cases are necessary to learn robotic surgery. [4]–[5].

Several meta-analysis studies have compared the short-term results among robotic, laparoscopic and open gastrectomy [6]–[11]. Our early experience with robotic gastrectomy was consistent with these meta-analysis; we found less operative blood loss and a shorter postoperative hospital stay compared with laparoscopic and open gastrectomy [12].

There have been few reports [5] comparing the learning curves between laparoscopic and robotic gastrectomy. This study was designed to compare the operative outcomes and learning curves between laparoscopic and robotic gastrectomy for gastric cancer patients, including cases that were performed during and after the surgeon’s learning curve.

## Materials and Methods

Laparoscopic gastrectomy has been performed since June 2006 at Taipei Veterans General Hospital. We have performed 97 laparoscopic gastrectomies; all of which were performed by two surgeons (W. -L. Fang, and J. -H. Chen). Collectively, these surgeons had experience of more than 100 cases of open gastrectomy before they began performing laparoscopic gastrectomy. Among the 97 laparoscopic gastrectomies, 73 patients were operated on by W. -L. Fang.

The da Vinci Si surgical system (Intuitive Surgical Inc., Sunnyvale, CA, USA) was introduced in our hospital in December 2009. Between August 2010 and August 2014, we performed 72 robotic gastrectomies for gastric cancer. All of the robotic surgeries were performed by a single surgeon (W. -L. Fang), who had experience with more than 30 cases of laparoscopic gastrectomy before performing robotic gastrectomy.

The clinicopathologic characteristics, the postoperative outcomes and learning curves were compared between patients who underwent laparoscopic and robotic gastrectomy for gastric cancer. We only enrolled minimally invasive surgery performed by a single surgeon, W. -L. Fang, in the present study. A total of 145 patients were enrolled in the study, including 73 patients in the laparoscopic group and 72 patients in the robotic group. The institutional review board at the Taipei Veterans General Hospital approved this study, and written informed consent was obtained from all of the patients. The pathological stages were classified according to the 7^th^ edition of the American Joint Committee on Cancer [13].

### Indication for laparoscopic and robotic gastrectomy

The indication for laparoscopic and robotic gastrectomy at our hospital was gastric cancer at a clinical stage lower than T3N1M0. Patients who were suitable for endoscopic mucosal resection or endoscopic submucosal dissection were referred to gastrointestinal endoscopists. Patients who had a history of gastric surgery were excluded from the study. Before surgery, the surgeons explained comprehensively both merits and demerits in the two operations to all patients. The decision for which type of surgical approach was made by the patients. The written informed consent was then provided to all patients.

All patients in the two groups were submitted to gastrectomy with at least D1+α (perigastric lymph nodes + No.7 lymph nodes) or D1+β(perigastric lymph nodes + No.7, 8, 9 lymph nodes) for early gastric cancer and D2 lymphadenectomy for advanced gastric cancer.

### Surgical procedures

#### Robotic gastrectomy

Under general anesthesia, the patient was placed in the reverse Trendelenburg position with the legs elevated approximately 15 degrees. The insertion of the trocars and docking with the robotic arms were mentioned in our previous study [12]. The ultrasonic shear was operated by the surgeon’s left hand, and the bipolar was controlled by the surgeon’s right hand. For patients receiving subtotal gastrectomy with robotic assistance, a 3- to 5-cm vertical incision was made at the upper abdomen. Since July 2013, we started to perform intracorporeal delta-shaped Billroth-I anastomosis with specimens removed from the umbilical wound, and there was no upper abdominal small vertical incision. Billroth I gastroduodenostomy, Roux-en-Y gastrojejunostomy, or uncut Roux-en-Y gastrojejunostomy was performed by the preference of the surgeon. For patients receiving a total gastrectomy, the same technique was used as in laparoscopic gastrectomy Roux-en-Y esophagojejunostomy was performed using a trans-oral anvil delivery system (EEA OrVil). For both subtotal and total gastrectomy, a close-suction drain was placed over the right subhepatic space. For total gastrectomy, an additional close-suction drain was placed over the left subphrenic space.

#### Laparoscopic gastrectomy

For laparoscopic gastrectomy, the overall operative process in the abdominal cavity is identical to that of robotic gastrectomy. The energy source, ultrasonic shears, was controlled by the right hand of the surgeon. The positions of the surgeon and assistant were different from robotic surgery, with the surgeon standing on the right side or between the legs of the patient, and the first assistant standing on the left side of the patient.

### Perioperative management

Nasogastric intubation was performed in the initial cases in the laparoscopic group, while no nasogastric tube intubation was applied in the robotic group or recent laparoscopic group. In our initial experience, the clinical pathway of laparoscopic gastrectomy was close to open gastrectomy, with water started on postoperative day 5 or day 6 and soft diet on postoperative day 9 to day 10. After accumulating more experience, water was usually started on postoperative day 3 or day 4, and a soft diet was started on postoperative day 5 to day 7. If no complication occurred, the patient was discharged.

### Statistical analysis

A statistical analysis was carried out using the software Statistical Package for Social Sciences 16.0 (SPSS; SPSS Inc., Chicago, IL, USA). Data are presented as means ± standard deviations (SDs). Independent Student’s t-test was used to compare the continuous variables among the two groups. Categorical data were compared using a chi-square test. Finally, *P* values less than 0.05 were considered to be statistically significant.

## Results

Table 1 shows the clinicopathologic characteristics between the laparoscopic and robotic gastrectomy groups. Patients in the robotic group were associated with more extracorporeal anastomosis with Roux-en-Y reconstruction, a higher percentage of D2 lymphadenectomy, and more medical costs compared to patients in the laparoscopic group. The retrieved lymph node number was similar between the two groups. There was no difference in the pathological T category, N category or the tumor-node-metastasis (TNM) stage between the two groups.

**Table 1:** Comparison of the clinicopathological differences between laparoscopic and robotic gastrectomy for gastric cancer.

	Laparoscopicgastrectomyn = 73	Roboticgastrectomyn = 72	*P* value
Age (years)	66.0±13.5	67.7±15.1	0.465
Gender (M/F)	42/31	40/32	0.868
Tumor size (cm)	3.3±1.6	3.2±1.5	0.688
BMI (kg/m^2^)	24.2±3.3	24.1±3.3	0.865
Resection extent			
Subtotal/total gastrectomy	63/10	64/8	0.802
Reconstruction method			0.001
Intracorporeal anastomosis (Billroth-I)	22 (30.1)	6 (8.3)	
Extracorporeal anastomosis (Roux-en-Y or uncut R-Y)	51 (69.9)	66 (91.7)	
Extent of lymphadenectomy			
D1+α/D1+β/D2	17/15/41	0/5/67	<0.001
Retrieved LN number	28.1±11.0	30.6±12.6	0.202
Pathological T category			0.757
T1/T2/T3/T4	49/10/11/3	52/9/10/1	
Pathological N category			0.228
N0/N1/N2/N3	55/10/3/5	50/8/10/4	
Pathological TNM stage			0.537
IA/IB	42/8	41/8	
IIA/IIB	12/6	15/1	
IIIA/IIB/IIIC	3/2/0	4/2/1	
Medical cost (US dollars)	2915.1±1341.4	5714.2±1591.7	<0.001

BMI: body mass index; LN: lymph node.

### Operative outcomes

Table 2 shows the operative outcomes of the two groups. The robotic group was associated with reduced operative blood loss (79.6±77.1 mL vs. 116.0±135.3 mL, *P* = 0.049) and longer operative time (357.9±107.8 min vs. 319.8±113.7 min, *P* = 0.040) compared to the laparoscopic group. There was no significant difference in the postoperative hospital stay, surgery and non-surgery related morbidity between the two groups. There was one mortality in the laparoscopic group related to duodenal stump leakage. There was one mortality in the robotic group related to gastrojejunostomy leakage.

**Table 2:** Operative outcomes of gastric cancer patients who underwent laparoscopic or robotic gastrectomy.

	LaparoscopicGastrectomyn = 73	RoboticGastrectomyn = 72	*P* value
Operative outcomes			
Operative time (min)	319.8±113.7	357.9±107.8	0.040
Operative blood loss (mL)	116.0±135.3	79.6±77.1	0.049
Postoperative hospital stay (day)	13.2±11.1	11.0±11.8	0.256
Surgical morbidity	6 (8.2)	9 (12.5)	0.587
Anastomosis leakage	3 (4.1)	3 (4.2)	
Anastomosis stenosis	1 (1.4)	0	
Delayed gastric emptying	4 (5.5)	5 (6.9)	
Chylous leakage	1 (1.4)	1 (1.4)	
Wound infection	0	1 (1.4)	
Intraabdominal abscess	2 (2.7)	2 (2.8)	
Intestinal obstruction	0	1 (1.4)	
Mortality	1 (1.4)	1 (1.4)	1.000

Some patients had more than one comorbidity.

Data were presented as mean±SD or n (%).

### Learning curves

**Figure 1:**
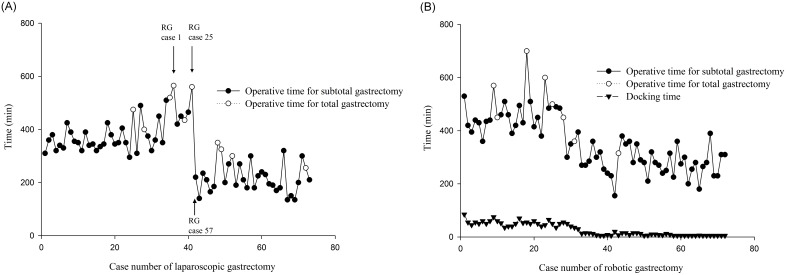
The operative time of laparoscopic and robotic gastrectomy decreased significantly after the learning curves. (A) The operative time of laparoscopic gastrectomy decreased after the 41th case. The arrows show the case number of robotic gastrectomy (RG) performed at the corresponding time of laparoscopic gastrectomy. (B) The operative time and docking time of robotic gastrectomy decreased after the 25th case.

Figure 1 shows the learning curves for laparoscopic and robotic gastrectomy. The operative time (299.9±71.3 min vs. 467.0±75.2 min, *P*<0.001) and docking time (13.6±13.3 min vs. 54.0±11.4 min, *P*<0.001) were significantly reduced in the recent robotic group (n = 47) compared to the initial robotic group (n = 25). The learning curve of robotic gastrectomy was defined as 25 cases as our previous study [12] and the series of Song et al [14].

In the laparoscopic group, the operative time (228.6±86.1 min vs. 393.9±70.8 min, *P*<0.001) and operative blood loss (53.4±49.5 mL vs. 164.9±159.6 mL, *P*<0.001) were significantly reduced in the recent laparoscopic group (n = 32) compared to the initial laparoscopic group (n = 41). Hence, we defined the learning curve as 41 cases in the laparoscopic group, which was close to a mean of 42 cases of learning curve in the study of Kim et al [15].

As shown in Table 3, we compared the differences in surgical performance and operative outcomes between the laparoscopic and robotic groups in the learning curve. The robotic group was associated with a longer operative time, less operative blood loss, a larger extent of lymphadenectomy and more medical costs compared with the laparoscopic group. As shown in Table 4, after the learning curve was overcome, the laparoscopic group was associated with more percentage of intracorporeal Billroth-I anastomosis, shorter operative time, and less medial cost compared to the robotic group. There were no significant differences in the surgical performance and operative outcomes between the two groups with regard to the operative blood loss, postoperative hospital stay, extent of gastric resection, extent of lymphadenectomy and retrieved lymph node number.

**Table 3:** Comparison of the surgical performance and operative outcomes between laparoscopic and robotic gastrectomy in the learning curve.

	Laparoscopic gastrectomy n = 41	Robotic gastrectomy n = 25	*P* value
Operative time (min)	393.9±70.8	467.0±75.2	<0.001
Extent of lymphadenectomy (D1+α/D1+β/D2)	17/14/10	0/4/21	<0.001
Retrieved LN number	26.2±11.5	31.0±13.7	0.124
Blood loss (mL)	164.9±159.6	92.0±88.0	0.040
Postoperativehospital stay (day)	15.5±12.7	12.7±17.4	0.453
Surgical morbidity	6 (14.6)	5 (20)	0.735
Surgical mortality	1 (2.4)	1 (4)	1.000
Medical cost (US dollars)	2787.6±1600	4259.5±1046.3	<0.001

LN: lymph node.

Data were presented as mean±SD or n (%).

**Table 4:** Comparison of the clinicopathological differences and operative outcomes between laparoscopic and robotic gastrectomy for gastric cancer after the learning curve.

	Laparoscopicgastrectomyn = 32	RoboticGastrectomyn = 47	*P* value
Age (years)	64.8±12.6	68.2±15.7	0.308
Gender (M/F)	15/17	29/18	0.250
Tumor size (cm)	3.6±1.7	3.2±1.4	0.207
BMI (kg/m^2^)	23.3±3.5	23.7±3.4	0.560
Resection extent			
Subtotal/total gastrectomy	28/4	44/3	0.432
Reconstruction method			0.001
Intracorporeal anastomosis	22 (68.8)	6 (12.8)	
(Billroth-I)			
Extracorporeal anastomosis	10 (31.3)	41 (87.2)	
(Roux-en-Y or uncut R-Y)			
Extent of lymphadenectomy			
D1+ β/D2	31/1	46/1	1.000
Retrieved LN number	30.6±10.0	30.±12.1	0.935
Operative time (min)	229.7±88.3	286.9±57.2	<0.001
Blood loss (mL)	52.7±50.2	73.0±70.8	0.176
Postoperative hospital stay (day)	10.4±7.9	10.2±7.3	0.873
Surgical morbidity	2 (6.3)	4 (8.5)	1.000
Medical cots (US dollars)	3083.6±890.8	6488.0±1256	<0.001
Pathological T category			0.182
T1/T2/T3/T4	16/4/9/3	34/4/8/1	
Pathological N category			0.318
N0/N1/N2/N3	20/5/3/4	33/4/8/2	
Pathological TNM stage			0.221
IA/IB	12/3	28/3	
IIA/IIB	7/5	10/1	
IIIA/IIB/IIIC	3/2/0	2/2/1	

BMI: body mass index; LN: lymph node.

## Discussion

The novelty of the present study is the surgeon’s initial experience of laparoscopic and robotic gastrectomy performed almost at the same period, and the results might be helpful to identify the effect of robotic gastrectomy on learning curve of laparoscopic gastrectomy for a beginning surgeon.

For the treatment of advanced gastric cancer, D2 lymph node dissection has been shown to have survival benefits over D1 lymphadenectomy [16]. However, the technical threshold of lymph node dissection during laparoscopic gastrectomy remains high and requires a long learning curve for surgeons who are accustomed to performing open gastrectomy. With the aid of robotic instruments, robotic gastrectomy can shorten the learning period of lymph node dissection and enable the surgeon to perform D2 dissection more easily than laparoscopic gastrectomy, and these surgeons will be able to perform extended lymph node dissections more easily when they return to laparoscopic gastrectomy.

We have started to perform intracorporeal Billroth-I anastomosis since July 2013. Before the 51th case in laparoscopic gastrectomy and the 66th case in robotic gastrectomy, we perform extracorporeal anastomosis with Roux-en-Y. It is of course that extracorporeal anastomosis with Roux-en-Y takes a longer time than intracorporeal Billroth-I anastomosis, which will affect the learning curves both in laparoscopic and robotic gastrectomy. However, the learning curves in both groups decrease before starting intracorporeal anastomosis. It seems that the method of anastomosis might not be the main cause of shortening the operative time, and the surgeon’s experience might play a more important role.

Our results showed that the operative time was longer in the robotic group even after the learning curve. As shown in Table 4, after the learning curve, the laparoscopic group was associated with more intracorporeal Billroth-I anastomosis than the robotic group. Both extracorporeal anastomosis and additional docking time might prolong the operative time in the robotic group. However, there is a trend of decreasing operation time after learning curve in both groups. We believe that after accumulating more experience and similar type of anastomosis, the operative time will decrease gradually and become more similar between the two groups.

Kim et al [5] reported that the experience of laparoscopic surgery could affect the learning process of robotic gastrectomy. However, for the beginning surgeon, could the experience of robotic surgeon have impact on the learning process of laparoscopic gastrectomy? Our data showed the operative time increased according to time sequence between the 25th to the 41th case of laparoscopic gastrectomy and dramatic shortening of the operation time after the 41th case. It is very interesting and what is the reason that could explain the dramatic change of the learning curve. First of all, the two patients with the longest operative time had a high BMI (>30), and one of them with the longest operative time had a huge inflammatory pseudotumor over right lobe of liver and a large lateral segment of liver, which made the operative exposure more difficult and prolonged the operative time. Moreover, we started to perform D2 lymphadenectomy since the 25th case of laparoscopic gastrectomy, which might increase the operative time. Between the 35th and 41th cases of laparoscopic gastrectomy, we started and performed 25 cases of robotic gastrectomy. The interval between the 41th and 42th cases of laparoscopic gastrectomy was 10 months, and we performed 32 cases of robotic gastrectomy during this period. Compared with laparoscopic gastrectomy, it is easier to perform D2 lymphadenectomy in robotic gastrectomy for the beginning surgeon. The reason why patients chose robotic gastrectomy instead of laparoscopic gastrectomy during this period might be influenced by the preoperative explanation of the surgeon because he was still in the learning period of D2 lymphadenectomy in laparoscopic gastrectomy. Surprisingly, as shown in Figure 1, the operative time of laparoscopic gastrectomy decreased dramatically after the 41th case. Decreasing of the operative time for both subtotal and total gastrectomy was observed after the learning curves of laparoscopic and robotic gastrectomy. Our results showed that the experience of robotic gastrectomy could contribute to the overcoming of the learning curve of laparoscopic gastrectomy for the beginning surgeon.

Even with support from the National Health Insurance, patients in Taiwan who undergo robotic gastrectomy must pay more than patients who undergo laparoscopic gastrectomy. In the learning curve of robotic gastrectomy, patients who undergo robotic gastrectomy need to pay nearly 1.5 times as patients who undergo laparoscopic gastrectomy ($4259.5±1046.3 vs. $2787.6±1600). The standard charging criterion was set up later after the learning curve in our hospital, and patients who undergo robotic gastrectomy at present need to pay nearly two to three times as patients who undergo laparoscopic gastrectomy ($6488.0±1256 vs. $3083.6±890.8). Surgeons may be more comfortable and experience less fatigue while performing robotic gastrectomy compared to laparoscopic gastrectomy, and the patient may receive limited benefits from robotic gastrectomy. Future studies should assess factors related to surgeon’s benefit including surgeon’s fatigue and comfort during operation. This difference presents an ethical dilemma that should not be ignored. Patients have the right to choose which operative approach they want, but surgeons should honestly and objectively explain both the surgeon’s and the patient’s benefits associated with each operative method, along with the operative risks to the patients before surgery.

The postoperative hospital stay was longer in the present study than other series. This might be because the clinical pathway for the initial experience of laparoscopic group was similar to that used with the open gastrectomy; water intake on postoperative day 5 or day 6, and soft diet on postoperative day 9 to day 10. We started to let patients try water and start liquid diet earlier as we gained more experience. At present, the time to try water and start a liquid diet is 3–4 days after surgery; and a soft diet is started on postoperative day 5 to day 7. If no complication occurred, the patient is discharged within 10 days after surgery in both laparoscopic and robotic groups. However, the mean postoperative hospital stay in the present study was 10.4 days for the laparoscopic group and 10.2 days for the robotic group even after the learning curve. For patients after the learning curve, most of the patients in the laparoscopic (83%) and robotic group (84%) discharged within 10 days after operation. The reasons for prolonged hospital stay in the two groups are due to surgical morbidity, including delayed gastric emptying, intestinal obstruction, and esophagojejunostomy leakage. In the future, we will try to decrease the surgical morbidity for minimizing the postoperative hospital stay.

In conclusion, the operative outcomes between laparoscopic and robotic gastrectomy become more similar as the surgeon accumulates experience. The experience of robotic gastrectomy could affect the learning process of laparoscopic gastrectomy. Long-term follow-up and prospective randomized studies are required to compare the oncological outcomes and quality of life between laparoscopic and robotic gastrectomy patients.
